# Persistence of severe global inequalities in the burden of Hypertension Heart Disease from 1990 to 2019: findings from the global burden of disease study 2019

**DOI:** 10.1186/s12889-023-17573-9

**Published:** 2024-01-06

**Authors:** Mengkai Lu, Dongxiao Li, Yuanlong Hu, Lei Zhang, Yuan Li, Zhiyuan Zhang, Chao Li

**Affiliations:** 1 Innovation Research Institute of traditional Chinese Medicine, Shandong University of Traditional Chinese Medicine, Jinan, 250355 China; 2 Experimental Center, Shandong University of Traditional Chinese Medicine, Jinan, 250355 China; 3 First Clinical Medical College, Shandong University of Traditional Chinese Medicine, Jinan, 250355 China; 4 College of Traditional Chinese Medicine, Shandong University of Traditional Chinese Medicine, Jinan, 250355 China

**Keywords:** Hypertensive heart disease, Global disease burden 2019, Health inequality, DALYs

## Abstract

**Aims:**

Assessing the global burden and health inequalities of Hypertension Heart Disease (HHD) during the period from 1990 to 2019.

**Methods:**

Secondary analysis of the Global Burden of Disease (GBD) study in 2019, focusing on the burden of diseases, injuries, and risk factors worldwide. Disability-Adjusted Life Years (DALYs) data related to HHD are extracted from the 2019 GBD. Inequality Slope Index (SII) and Concentration Index are calculated to assess health inequalities across regions and countries.

**Results:**

The total DALYs for HHD reached 21.51 million, demonstrating a substantial increase of 54.25% compared to the figures recorded in 1990, while the age-standardized DALY rates per 100,000 population for HHD in 2019 showed a notable decline to 268.19 (95% UI 204.57, 298.07), reflecting a significant decrease of 26.4% compared to the rates observed in 1990. The DALYs rate of hypertensive heart disease increases with age. Countries with moderate SDI accounted for 38.72% of the global burden of HHD in terms of DALYs. The highest age-standardized DALY rates (per 100,000) are predominantly concentrated in underdeveloped areas. In 1990 and 2019, the SII (per 100,000 population) for DALYs were − 121.6398 (95% CI -187.3729 to -55.90684) and − 1.592634 (95% CI -53.11027 to 49.925) respectively. The significant decline suggests a reduction in the inequality of age-standardized burden of HHD between high-income and low-income countries during this period.

**Conclusion:**

The unequal prevalence of HHD across different populations can hinder the achievement of the “health for all” objective. Persistent disparities in HHD have been observed globally over the past thirty years. It is crucial to prioritize efforts towards reducing avoidable health inequalities associated with hypertension-related heart disease, particularly in low-income and middle-income countries.

## Introduction

HHD is a medical condition that is characterized by a range of pathological alterations in the heart, primarily affecting the left ventricle. It develops as a consequence of prolonged high blood pressure and is associated with several manifestations such as myocardial hypertrophy, arteriosclerosis, and cardiac arrhythmias [1, 2]. The clinical presentation of HHD varies, ranging from no symptoms or mild chest discomfort and palpitations to breathlessness, heart failure, and potentially even sudden cardiac death [3]. HHD is a disease caused by high blood pressure, and is a key underlying mechanism for the incidence and mortality of cardiovascular diseases [4]. As the fourth most common cause of cardiovascular and cerebrovascular fatalities, hypertensive heart disease ranks just below ischemic heart disease, stroke, and cardiomyopathy [5].

In this study, our analysis was based on the comprehensive dataset of GBD 2019. The primary objectives were as follows: (1) to evaluate the disparities in health outcomes associated with hypertensive heart disease, taking into account the Socio-demographic Index (SDI); (2) to examine the temporal patterns of health inequalities related to hypertensive heart disease between 1990 and 2019. The overall goal of this study is to provide comprehensive insights and help prioritize and allocate healthcare resources to alleviate the health inequalities and disease burden of HHD globally across countries.

## Methods

### Data sources

We downloaded the Global Burden of Disease Study 2019 data on annual Disability-Adjusted Life Years (DALYs) associated with HHD at the global, regional, and national levels, as well as the Socio-demographic Index (SDI) data for population and social demographic indices. The HHD is defined by the International Classification of Diseases (ICD) -9 codes 402-402.9 and ICD-10 codes I11-I11.9 [6]. The health burden of HHD was assessed by utilizing existing data, dividing it by age and gender, for 204 countries or regions [7, 8]. The Global Burden of Disease study (GBD) utilizes a comprehensive meta-regression framework to address the scarcity and heterogeneity of data. It provides a range of interconnected indicators to assess the burden of disease, such as incidence, prevalence, mortality, years of life lost (YLLs), years lived with disability (YLDs), and DALYs, with DALYs being widely used as a summary measure of health. DALYs serves as a composite indicator that quantifies the loss of healthy life years, taking into account both the quantity and quality of life over time. SDI is a composite indicator that encompasses the following components: national-level per capita income, average educational attainment of the population aged 15 and above, and total fertility rate among women under the age of 25. The SDI ranges from 0 (high fertility rate, low education, low income) to 1 (low fertility rate, high income, high education). Each GBD location is assigned to an individual SDI group based on its SDI value in 2019 [9, 10].

The disease burden of HHD at the global, regional, and national levels was evaluated using descriptive analysis. The number of cases per 100,000 population, total DALYs, crude DALY rates, and age-standardized DALY rates were calculated. We utilized scatter plots to depict the gender-stratified trends in the global burden of each subtype of HHD from 1990 to 2019. For each age group, we presented the burden distribution in 1990 and 2019 using age-sex pyramids, and displayed specific cause burden with stacked bar charts. We compared the disease burden of hypertensive heart disease across different regions globally. Additionally, we employed disease mapping to visualize the total DALYs and age-standardized DALY rates per 100,000 population at the national level.

### Measurement health inequalities

Total DALYs and age-age standardized DALY rates were extracted for inequality analysis. As per the recommendations of the World Health Organization, two standard measures, namely Slope Index of Inequality (SII) and Concentration Index, were used to assess both absolute and relative income-related inequalities between countries [11]. The SII represents the slope of the regression line that correlates the country-level age-standardized DALY rate (ASDR) associated with HHD with the weighted ranking of each country. To adjust for variations in burden levels, the SII is divided by the global ASDR, yielding the Relative Index of Inequality (RII). The CI is utilized to assess the relative disparity in the burden of HHD among countries by fitting the Lorenz concentration curve based on cumulative DALYs and cumulative population. The CI is a numerical integration of the area under the curve, ranging from − 1 to 1. A negative CI value indicates a higher concentration of HHD burden among populations residing in countries with lower SDI.

### Patient and public involvement

No patients or members of the public were directly involved in this study. There are no plans to involve patients or the public in the dissemination of the results.

## Results

### Global burden of HHD

According to GBD 2019, in 2019, the number of deaths due to HHD was 1.1567 million (95% UI 0.8598 million to 1.2786 million) among the measured population across all age groups. This accounted for 2.046% of the burden of all 369 diseases, an increase of 76.63% (95% UI 62.06–74.49%) compared to 1990. Based on Table 1, the data reveals that in 2019, the burden of HHD was significant. The total DALYs for HHD reached 21.51 million, demonstrating a substantial increase of 54.25% compared to the figures recorded in 1990. The DALYs rates per 100,000 population stood at 277.97 (95% UI 211.96, 308.89), indicating a moderate increase of 6.65% since 1990. However, it is noteworthy that the age-standardized DALY rates per 100,000 population for HHD in 2019 showed a notable decline to 268.19 (95% UI 204.57, 298.07), reflecting a significant decrease of 26.4% compared to the rates observed in 1990. Compared to 1990, the DALYs (Disability-Adjusted Life Years) for hypertensive heart disease attributable to high body-mass index and alcohol in 2019 showed the highest increase rates among the included risk factors, with rises of 145.34% and 68.4%, respectively.

**Table 1:** Global burden of hypertensive heart disease and the specific causes in 1990 and 2019

	DALYs			DALYs rates			Age-standardised DALY rates		
	1990 (million)	2019 (million)	Change (%)	1990 (per 100,000)	2019 (per 100,000)	Change (%)	1990 (per 100,000)	2019 (per 100,000)	Change (%)
All risk factors	13.94(11.31,15.65)	21.51(16.40,23.90)	54.25	260.64(211.45,292.53)	277.97(211.96,308.89)	6.65	364.55(297.73,406.70)	268.19(204.57,298.07)	-26.4
Lead exposure	1.31(5.90,2.68)	1.77(0.66,3.85)	35.24	24.46(11.04,50.02)	22.87(8.58,49.70)	-6.50	33.36(14.33,69.08)	22.04(8.09,47.48)	-0.34
Alcohol use	1.22(0.77,1.72)	2.05(1.27,2.97)	68.4	22.79(14.40,32.22)	26.53(16.48,38.41)	16.44	31.18(19.67,44.16)	25.34(15.75.36.68)	-18.71
High systolic blood pressure	13.94(11.31,15.65)	21.51(16.40,23.90)	54.25	260.64(211.45,292.53)	277.97(211.96,308.89)	6.65	364.55(297.73,406.70)	268.19(204.57,298.07)	-26.43
High body-mass index	3.55(1.83,5.81)	8.70(5.48,12.57)	145.34	66.32(34.23,108.68)	112.50(70.80,162.44)	69.63	90.20(46.10,148.89)	106.88(66.08,156.44)	18.50
Diet high in sodium	2.92(0.94,6.51)	3.51(0.84,8.80)	20.28	54.50(17.58,121.65)	45.32(10.89,113.67)	-16.83	73.96(23.12,171.00)	43.07(10.17,109.39)	-41.77
High temperature	-0.03(-0.46,0.36)	0.04(-0.35,0.41)	-216.19	-0.57(-8.68,6.73)	0.46(-4.51,5.26)	-180.34	-0.77(-11.58,8.90)	0.44(-4.24,4.94)	-157.10
Low temperature	0.89(0.56,1.21)	1.19(0.68,1.64)	33.61	16.71(10.39,22.53)	15.43(8.72,21.15)	-7.62	23.63(14.84,31.82)	15.04(8.54,20.60)	-36.37

*Central estimates with 95% uncertainty intervals.

DALYs, disability-adjusted life-years.

**Fig. 1:**
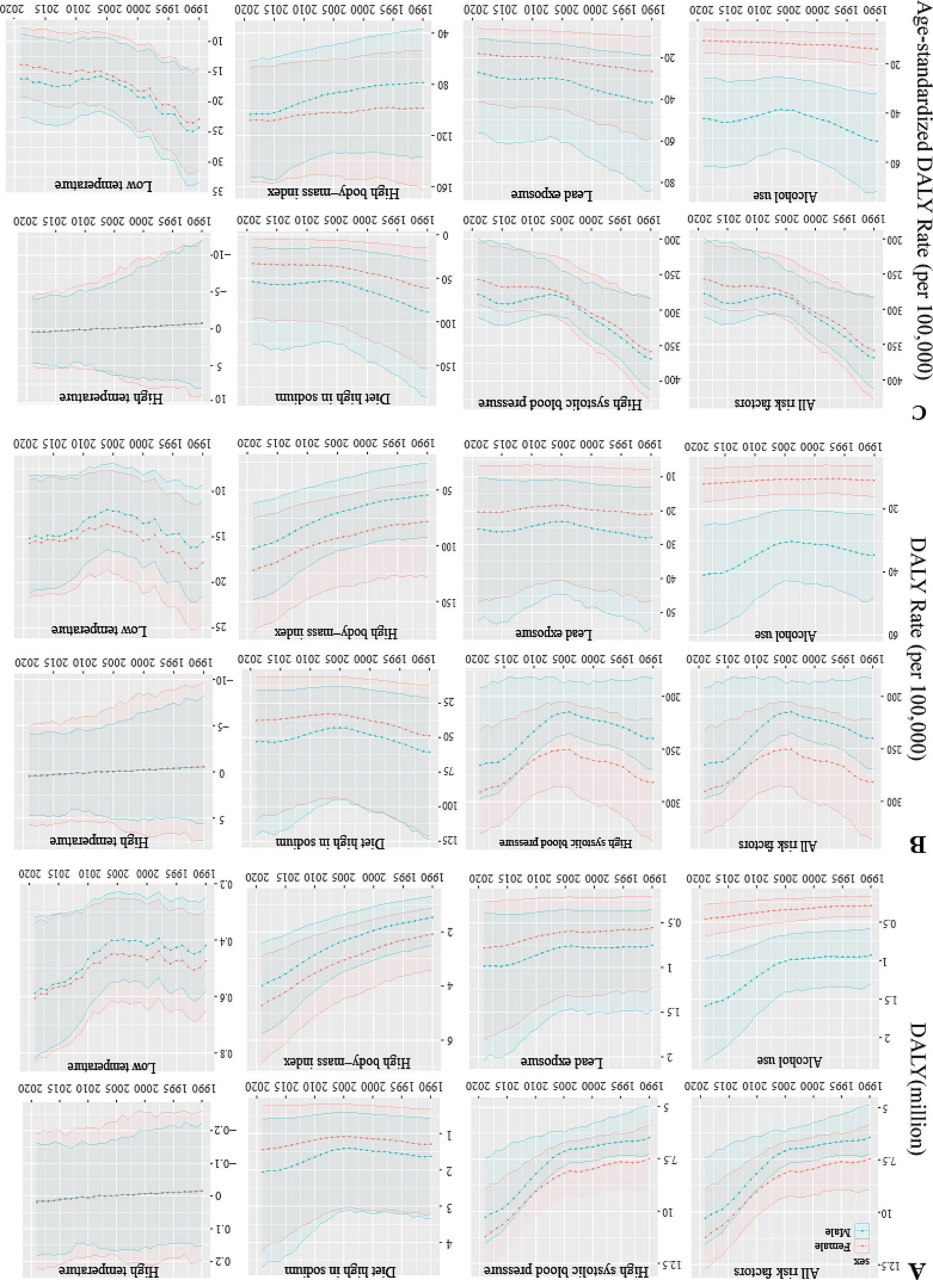
Global burden due to kinds of HHD by sex

**Fig. 2:**
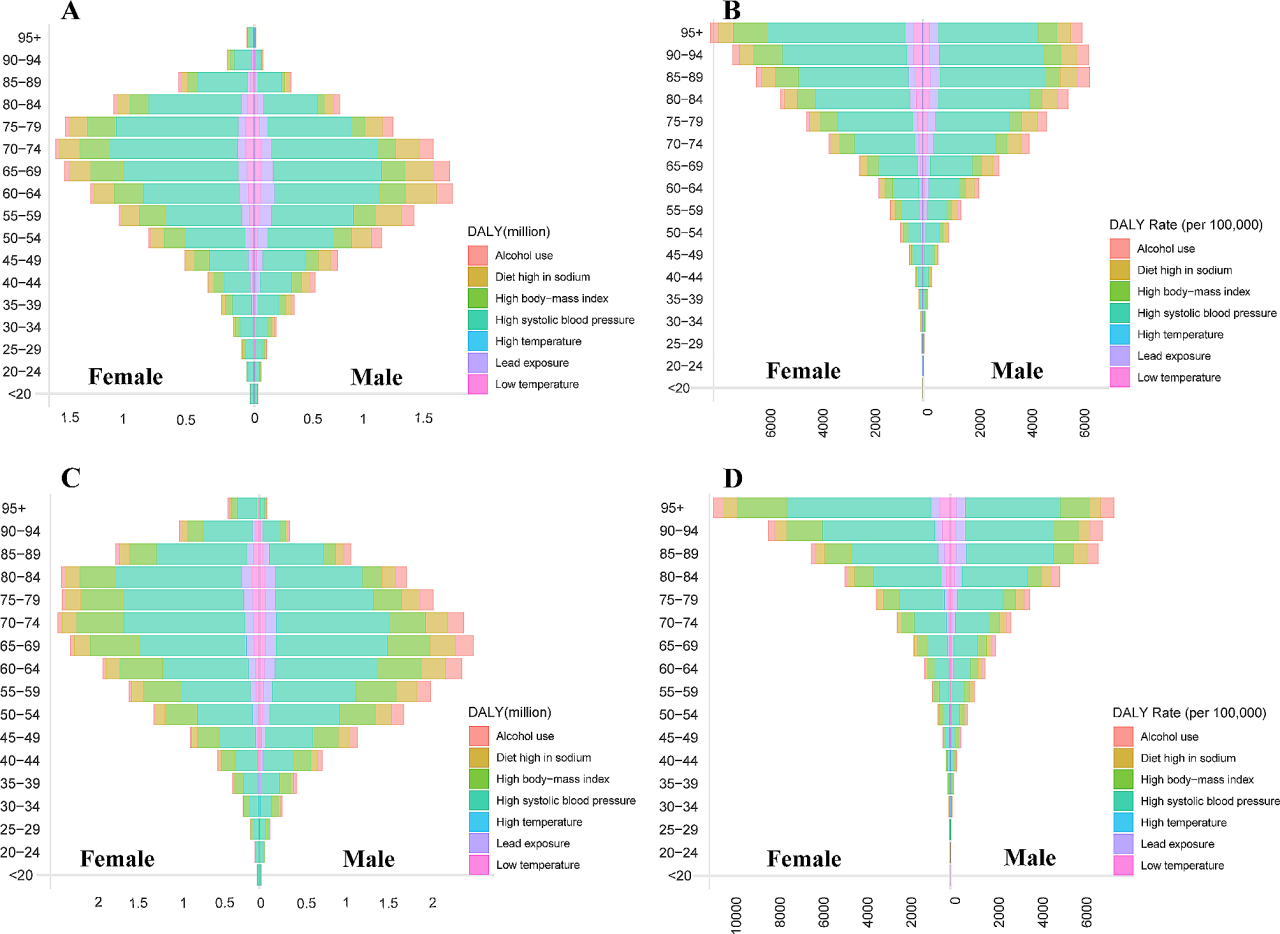
The age distribution of the burden of HHD by sex in 1990 (**A**, **B**) and 2019 (**C**, **D**) expressed in DALYs (**A**, **C**) and DALY rates (**B**, **D**)

Among the seven risk factors for hypertensive heart disease included in GBD 2019, women bear a heavier burden of the disease compared to men, even after adjusting for age (Fig. 1 and Fig. 2). In both 1990 and 2019, the age-specific burden of hypertensive heart disease was highest in the 70–74 age group (Fig. 2A, C). After adjusting for population size within each age group, it was found that the DALYs rate of hypertensive heart disease increases with age (Fig. 2B, D).

### The spatial distribution of the burden of Hypertensive Heart Disease (HHD)

**Fig. 3:**
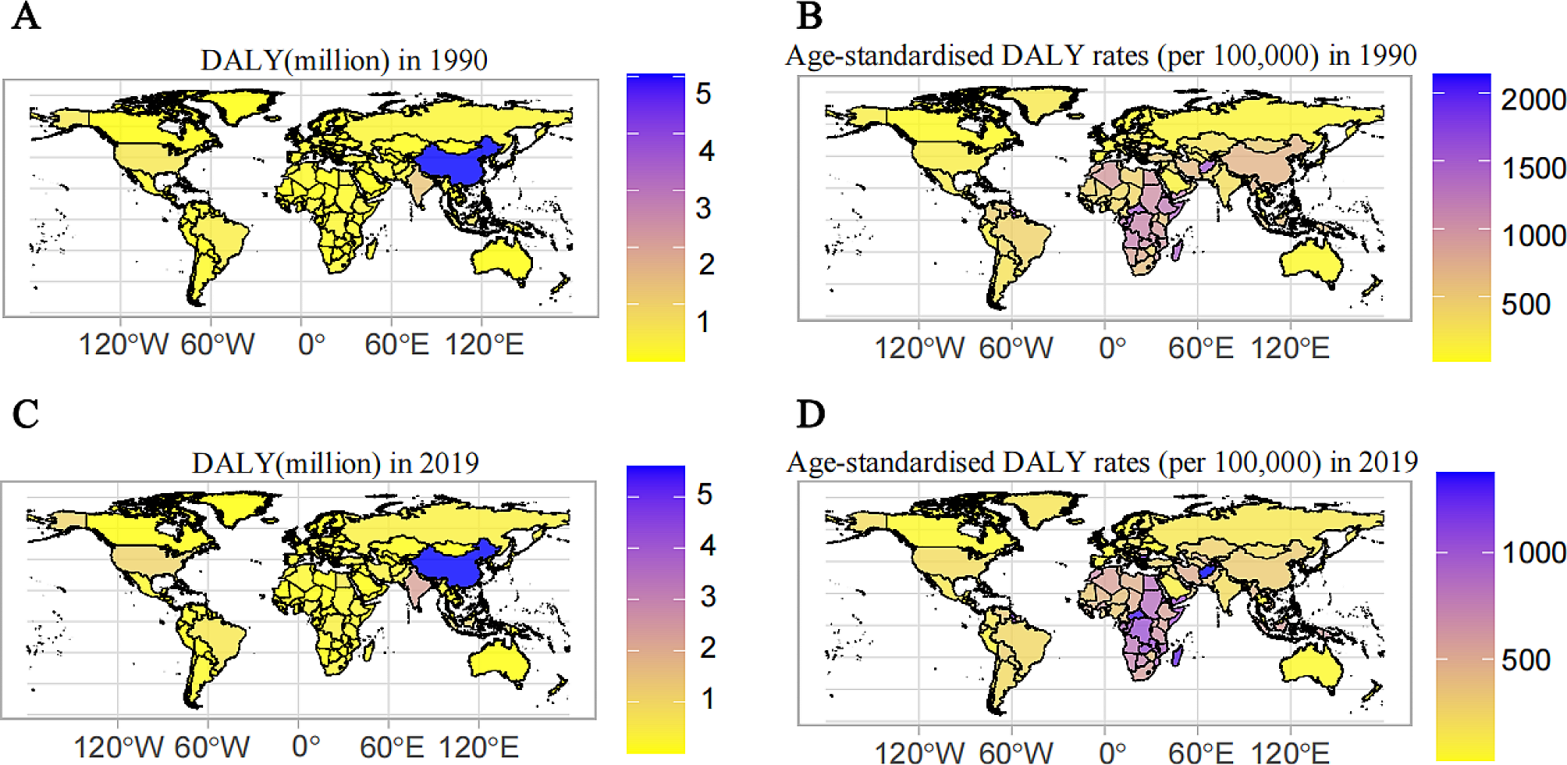
Spatial distribution of the burden of HHD, in terms of DALY in all age

**Fig. 4:**
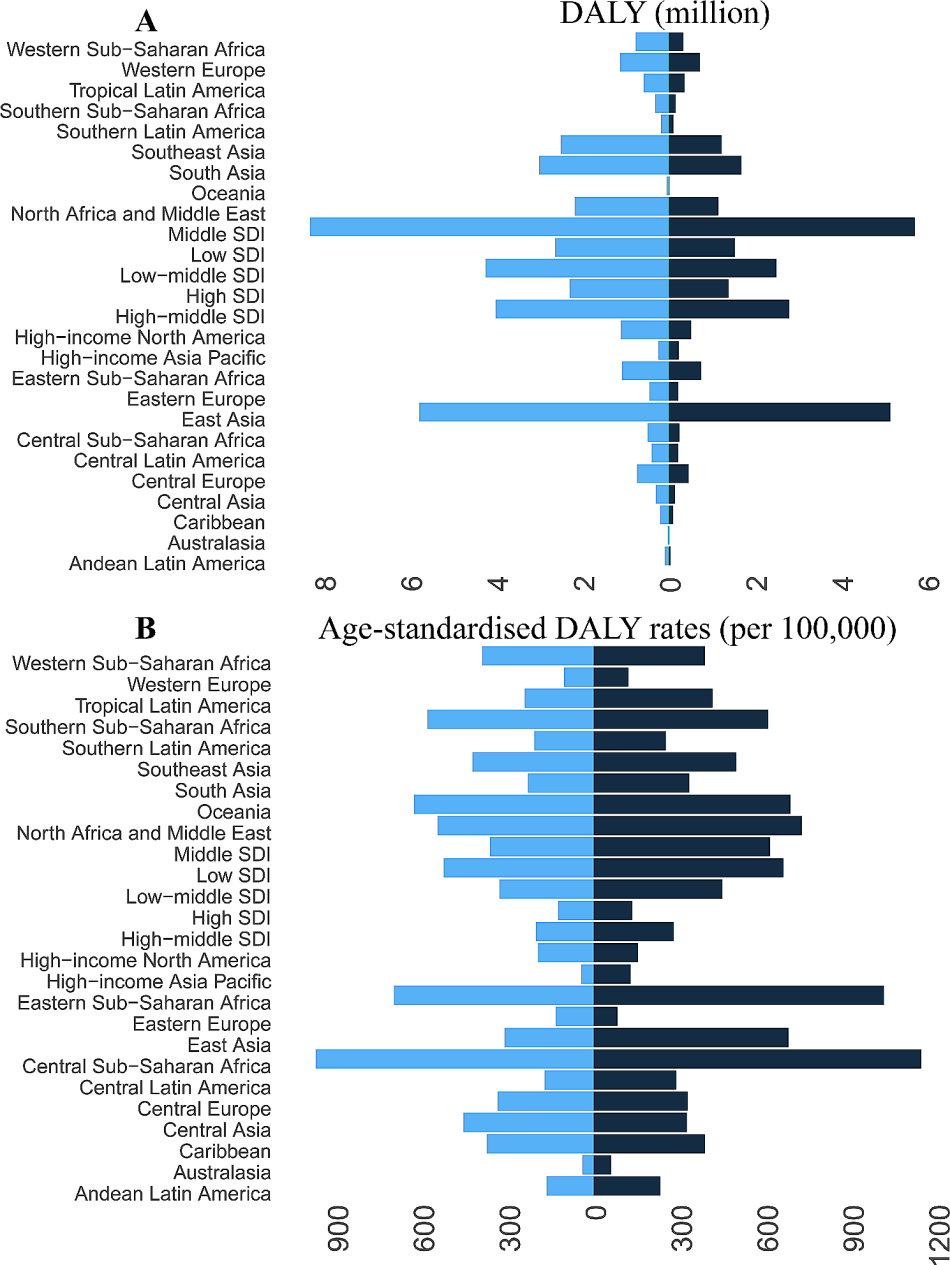
Change in burden of HHD by region, 1990 vs. 2019

In 2019, countries with moderate Socio-Demographic Index (SDI) accounted for 38.72% of the global burden of HHD in terms of DALYs. East Asia, South Asia, and Southeast Asia emerged as the regions most heavily impacted by HHD globally in 2019 (Fig. 4). In 2019, the burden of HHD was most severe in five countries at the national level. China (5.59, 95% UI 3.88 to 6.53 million), India (2.26, 95% UI 1.52 to 3.0 million), Indonesia (1.16, 95% UI 0.65 to 1.43 million), the United States of America (1.08, 95% UI 0.72 to 1.17 million), and Brazil (0.56, 95% UI 0.50 to 0.77 million) were identified as the countries with the highest HHD burden in terms of DALYs (Fig. 3C).

Compared to 1990, there has been a substantial rise in the burden of HHD measured in DALYs. By 2019, DALYs for HHD had increased by 54.25%. However, the spatial distribution pattern has remained consistent (Fig. 3A **and C**). This consistency indicates that the trend of HHD burden growth among different regions and countries remained relatively stable during this period, without significant spatial distribution disparities.

Moreover, regions characterized by the highest age-standardized DALY rates (per 100,000) are predominantly concentrated in underdeveloped areas. The top five regions with the highest age-standardized DALY rates are Central Sub-Saharan Africa, Central Sub-Saharan Africa, Oceania, Southern Sub-Saharan Africa, and North Africa and the Middle East. From 1990 to 2019, the age-standardized DALY rates of HHD showed a declining trend in most GBD regions and countries. Over this time period, the countries with the highest age-standardized DALY rates were predominantly concentrated in Asia and Africa (Fig. 3B **and D**).

### Cross-national HHD health inequality

**Fig. 5:**
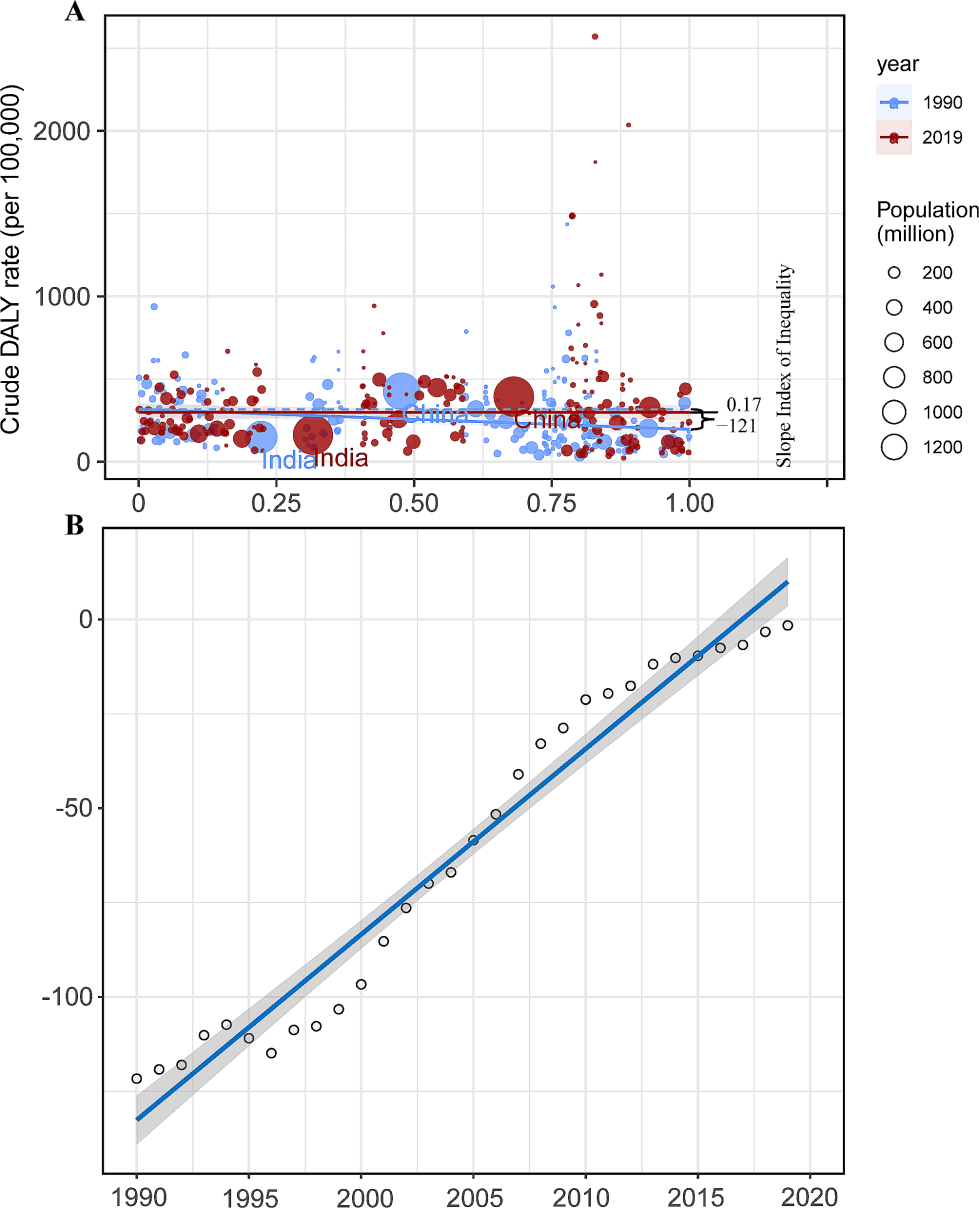
SII analysis. (**A**) Absolute income-­related healthy inequality in HHD burden, presented using regression lines, 1990 vs. 2019. (**B**) Trendline demonstrates the trend in SII from 1990 to 2019

**Fig. 6:**
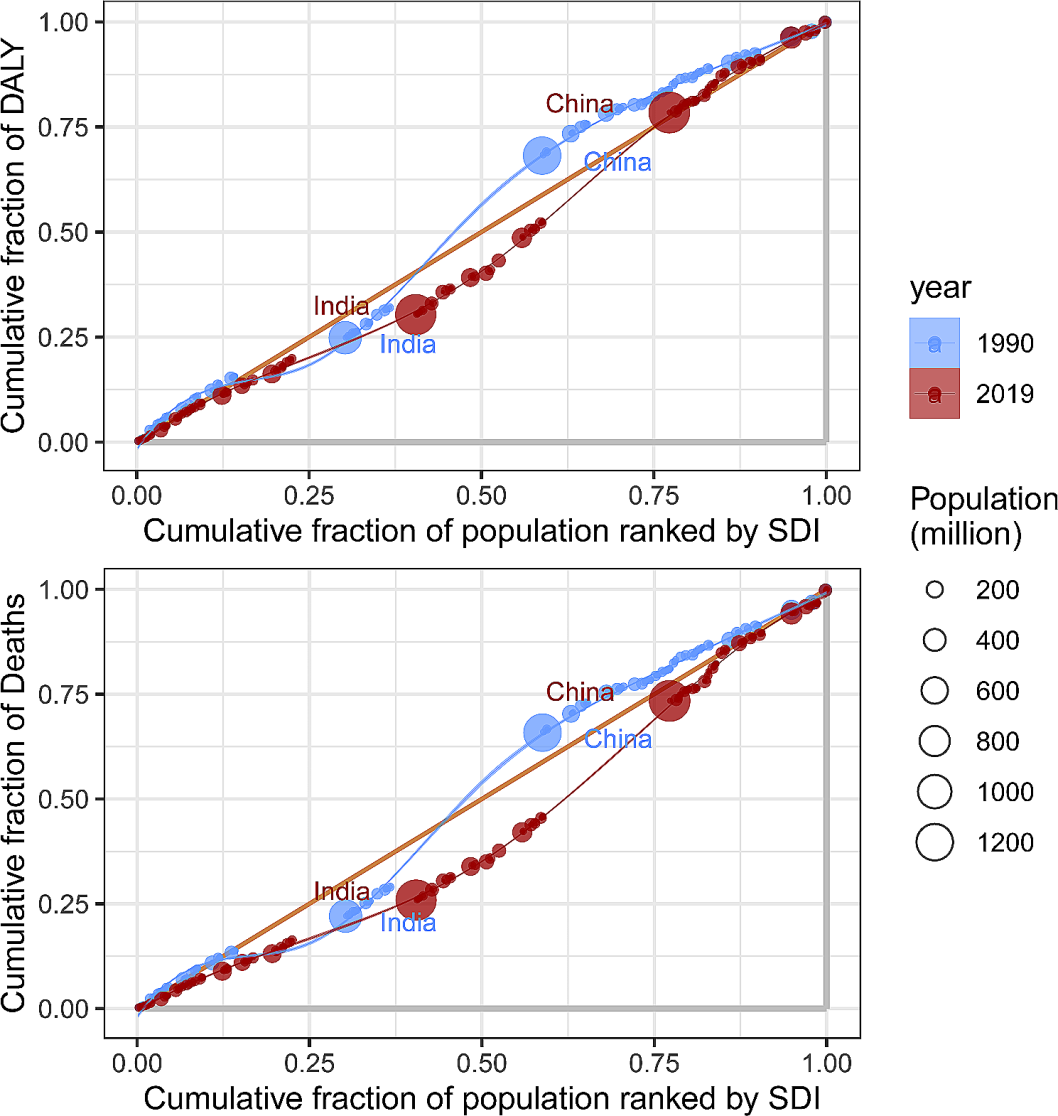
Concentration index analysis. (**A**) Relative income-­related healthy inequality in HHD burden, presented using concentration curves, 1990 vs. 2019. (**B**) Trendline demonstrates the trend in concentration index from 1990 to 2019

In 1990 and 2019, the SII (per 100,000 population) for DALYs were − 121.6398 (95% CI -187.3729 to -55.90684) and − 1.592634 (95% CI -53.11027 to 49.925) respectively, indicating a negative correlation between age-standardized DALY rates and SDI index (Fig. 5). This significant decline suggests a reduction in the inequality of age-standardized burden of HHD between high-income and low-income countries during this period. Between 1990 and 2019, the concentration index for DALYs and Deaths has shown a declining trend. Although the inequality in the burden of hypertensive heart disease has decreased regionally between poor and wealthy countries, inequality still persists. This indicates that while the wealth gap has narrowed in some regions, global inequality in hypertensive heart disease remains a persistent issue (Fig. 6).

## Discussion

The secondary analysis of the GBD 2019 research data offers a comprehensive global overview of the health burden attributed to HHD. It specifically sheds light on the cross-national health disparities resulting from socioeconomic inequalities. Our findings demonstrate that the burden of DALYs associated with HHD is primarily concentrated in economically disadvantaged and underdeveloped regions, affecting predominantly male populations, as well as middle-aged and elderly individuals. In addition, although there has been a decrease in the global age-standardized burden of HHD, the overall burden continues to rise, highlighting persistent health inequalities associated with HHD. Research indicates that the increase in the number of DALYs associated with HHD globally can be attributed to both population growth and aging [12, 13]. According to estimates, the global population has grown from 5.35 billion (95% UI 5.24 to 5.46 billion) in 1990 to 7.74 billion (95% UI 7.48 to 7.99 billion) in 2019 [10]. In England and Wales, the elderly population accounted for 18.6% of the total population in 2021. It is estimated that by 2030, the elderly population will surpass 1 billion people [14, 15]. The age-standardized DALY rates reflect the trends in the global burden of hypertensive heart disease (HHD) from 1990 to 2019, indicating a consistent decline in HHD burden (age-standardized) over the past 28 years. HHD is considered an age-related disease, and both individual and global disease burdens have increased with the aging of patients and populations.

It is estimated that globally, approximately 1.39 billion adults (aged ≥ 20) are living with hypertension, with 694 million men and 694 million women affected, in 2010 [16]. The number of individuals with hypertension in low-income and middle-income countries is nearly triple that of high-income countries. In terms of healthcare expenditure on hypertension, the global burden was estimated to be 1.44 billion US dollars in 2010, and it is projected to exceed 1.6 billion US dollars by 2025 [16]. Hypertension is a major risk factor for cardiovascular disease [17, 18]. In 2019, the number of deaths among young people globally due to cardiovascular diseases related to hypertension was 640,239, representing a 43.0% increase compared to 1990 [19]. These results align with our findings.

Health inequality monitoring utilizes data on health inequalities, which refer to significant differences in health outcomes among different population subgroups. It provides information for policies and programs aimed at addressing health inequalities, including unfair, avoidable, or remediable health disparities [20, 21].

In terms of age distribution, the age-standardized burden of HHD is closely associated with increasing age, consistent with previous reports. As HHD has been identified as an age-related disease, its global disease burden increases with both the aging of patients and the population as a whole [1, 22].

In terms of inter-country inequality, the disease distribution map and cross-regional comparisons reveal that the burden of HHD is closely associated with the socio-economic index, consistent with previous reports [23]. Regarding the SDI index, the DALYs for HHD are highest in regions with moderate SDI and lowest in regions with high SDI. The age-standardized DALYs rates are highest in low SDI regions and lowest in high SDI regions.

Although the aforementioned results are precise, they do not provide the optimal comparability for measuring and monitoring inequalities. To address this, we calculated the Slope Index of Inequality (SII) and Concentration Index based on the SDI index. We found that in 2019, the burden of HHD showed a negative correlation with the socio-economic level. Over the past 30 years, this inequality has consistently decreased in terms of the Slope Index of Inequality (SII), but has remained at a moderately high level in relative measures (Concentration Index).

This study has several limitations. Firstly, the limited data collection in economically underdeveloped regions may result in an underestimation of the true burden of HHD. Secondly, the lack of clinical information on HHD in the GBD 2019 study and reliance on secondary analysis of available data are noteworthy. Thirdly, the use of aggregated national-level data instead of regional-level data may introduce potential bias in estimating DALYs and can lead to geographical variations. Furthermore, the GBD study has taken several steps to improve the reliability and comparability of data, but in some parts of the world, health data on cardiovascular diseases remains extremely limited. The lack of data is a significant reason why no significant change in the prevalence or mortality of hypertensive heart disease could be detected in many regions. In countries with overestimated cardiovascular disease mortality, only limited data is available. Additionally, the GBD study includes an estimate of measurement error for each result, reported in the form of a 95% uncertainty interval (UI). In regions with wide UIs, the ability to detect time trends is also limited. Other sources of error in the GBD study may include regional patterns of clinical diagnosis of HHD, death code redistribution, selection of data sources, and the measurement of the Socio-Demographic Index. Furthermore, similar results (such as data related to the burden of HHD and its spatial distribution [24]) have already been published using data from the GBD 2019 database. While this study offers a global perspective on the socio-economic inequality of HHD burden, the conclusions may not be directly applicable to specific regions.

In summary, this study reveals that despite societal advancements, the burden of hypertensive heart disease (HHD) continues to be concentrated in countries with moderate SDI indices. To mitigate global inequalities arising from socio-economic disparities, it is crucial to ensure a more equitable utilization and allocation of healthcare resources. This entails prioritizing the needs of vulnerable populations, including the poor, elderly, and male individuals. By doing so, we can work towards reducing the disparities and promoting better health outcomes for all.

## Data Availability

The data used in this study came from a public database that everyone can access through the link provided in this article (https://vizhub.healthdata.org/gbd-results/).
